# Conducting ITO Nanoparticle-Based Aerogels—Nonaqueous One-Pot Synthesis vs. Particle Assembly Routes

**DOI:** 10.3390/gels9040272

**Published:** 2023-03-25

**Authors:** Samira Sang Bastian, Felix Rechberger, Sabrina Zellmer, Markus Niederberger, Georg Garnweitner

**Affiliations:** 1 Institute for Particle Technology, Technische Universität Braunschweig, Volkmaroder Str. 5, 38104 Braunschweig, Germany; 2 Laboratory for Multifunctional Materials, Department of Materials, ETH Zurich, Vladimir-Prelog-Weg 5, 8093 Zurich, Switzerland; 3 Fraunhofer Institute for Surface Engineering and Thin Films (IST), Bienroder Weg 54E, 38108 Braunschweig, Germany; 4 Laboratory for Emerging Nanometrology, Technische Universität Braunschweig, Langer Kamp 6A, 38106 Braunschweig, Germany

**Keywords:** indium tin oxide, conductive aerogel, nanoparticle-based gel, nonaqueous synthesis, transparent conductive oxide

## Abstract

Indium tin oxide (ITO) aerogels offer a combination of high surface area, porosity and conductive properties and could therefore be a promising material for electrodes in the fields of batteries, solar cells and fuel cells, as well as for optoelectronic applications. In this study, ITO aerogels were synthesized via two different approaches, followed by critical point drying (CPD) with liquid CO_2_. During the nonaqueous one-pot sol–gel synthesis in benzylamine (BnNH_2_), the ITO nanoparticles arranged to form a gel, which could be directly processed into an aerogel via solvent exchange, followed by CPD. Alternatively, for the analogous nonaqueous sol–gel synthesis in benzyl alcohol (BnOH), ITO nanoparticles were obtained and assembled into macroscopic aerogels with centimeter dimensions by controlled destabilization of a concentrated dispersion and CPD. As-synthesized ITO aerogels showed low electrical conductivities, but an improvement of two to three orders of magnitude was achieved by annealing, resulting in an electrical resistivity of 64.5–1.6 kΩ·cm. Annealing in a N_2_ atmosphere led to an even lower resistivity of 0.2–0.6 kΩ·cm. Concurrently, the BET surface area decreased from 106.2 to 55.6 m^2^/g with increasing annealing temperature. In essence, both synthesis strategies resulted in aerogels with attractive properties, showing great potential for many applications in energy storage and for optoelectronic devices.

## 1. Introduction

Transparent conducting oxides (TCOs) play an important role in modern electronic devices such as displays and smartphones. In the form of thin films, they are commonly used as transparent electrodes in light-emitting diodes (LED), liquid crystal displays (LCD), solar cells, smart electronics and chemical sensors [1,2,3,4,5,6]. As their name indicates, they offer the unique combination of high transmission in the near-infrared and visible wavelength range and an n-type electrical conductivity [7,8,9]. Despite the scarcity of In resources, doped indium oxide, in particular ITO with an In:Sn ratio of about 9:1, remains the most common TCO material due to its outstanding electrical conductivity and optical transmittance [10].

TCO layers on glass or plastic substrates can be generated through physical or chemical vapor deposition (PVD/CVD) techniques, sputtering, vacuum deposition or through wet chemical processing [5,6]. While the latter approach bears the advantage of low cost, it requires an annealing treatment to obtain a crystalline TCO film, which limits its application on polymeric substrates. An attractive alternative is the use of sols containing already crystalline TCO nanoparticles that can be applied via various coating techniques (spin, spray or dip coating) and lead to a conducting film after drying at relatively low temperatures [4,5,6,11].

In contrast to dense thin films, mesoporous structures enable a strong interaction with the surrounding medium and can act as electrodes in bulk heterojunction solar cells or fuel cells with the highest efficiency [12,13,14]. The use of an ITO aerogel is one way to overcome the challenge of connecting ITO nanoparticles into a conducting three-dimensional architecture [4,15]. Aerogels are solid networks with a defined mesoporous structure with a pore size of up to 100 nm and a porosity exceeding 80%, hence possessing a very high specific surface area and a low density [16,17]. Reports on the synthesis of conductive metal oxide aerogels are still rare [18]. A popular method for synthesizing ITO-based nanostructured gels is the sol–gel method using soft (e.g., surfactants or block copolymers) or hard templates (e.g., silica spheres), for which the removal of the respective template constitutes a difficult challenge [16]. Davis et al. demonstrated a template-free approach for the preparation of ITO aerogels in which the synthesis is based on a sol–gel process under the addition of epoxides, as previously reported for the preparation of monolithic iron oxide gel; however, low crystallinity and limited synthesis control had to be accepted [19,20].

One approach that has been extensively applied for the fabrication of aerogels of diverse metal oxides in recent years is the utilization of crystalline nanoparticles as pre-formed building blocks, which are arranged into a gel via induced coagulation, followed by CPD [14,15,21,22,23]. High crystallinity and purity, a narrow size distribution and good dispersibility in solvents are essential properties of the nanoparticles to achieve highly porous, homogeneous and functional aerogels [15,22]. A proven approach to prepare ITO nanoparticles with such properties is nonaqueous sol–gel synthesis [24], which offers relatively simple reaction parameters and high yields and is also suitable for scaling up [25]. This approach leads to uniform nanoparticles despite the absence of surfactants, since the solvent acts as a growth-controlling agent, in addition to its function as a reactant, and the doping level can also be easily adjusted [24,26,27]. Solvents such as BnOH or BnNH_2_ are commonly used for nonaqueous sol–gel synthesis, bearing a functional group that can control the growth kinetics and influence the particle morphology [26,28,29]. Stable colloidal dispersions of the ITO nanoparticles, which are required to achieve highly homogeneous gels, can be obtained in a post-synthetic stabilization procedure via the simple addition of organic ligands [30,31,32].

In this paper, we present the synthesis of conducting ITO aerogels obtained via two different synthesis strategies. First, the direct preparation of aerogels via a nonaqueous one-pot sol–gel synthesis in BnNH_2_ is shown, resulting in a blue cylindrical gel that could be easily converted into an aerogel via solvent exchange and supercritical drying. Alternatively, ITO nanoparticles were prepared by nonaqueous sol–gel synthesis in BnOH via an autoclave-assisted route and a microwave-assisted route, and were subsequently assembled into aerogels in a particle-based approach after functionalization with 2-[2-(2-methoxyethoxy)ethoxy]acetic acid (MEEAA). The aerogels obtained from these different approaches were compared with respect to a number of characteristics, especially their structural properties, as well as their electrical resistivity before and after annealing. Consequently, the influence of the synthesis approach on the properties of the aerogels is elucidated, allowing for a direct comparison of different strategies for the formation of particle-based aerogels for the first time. Calcination resulted in an improvement in the electrical conductivity by more than two orders of magnitude while retaining a large surface area and homogeneous porosity throughout the entire volume. This study demonstrates that highly crystalline ITO aerogels with interesting electrical properties can be achieved via a simple and scalable process.

## 2. Results and Discussion

Three different types of ITO aerogels were obtained by two different synthesis strategies. The first strategy is nonaqueous one-pot synthesis with In(acac)_3_ and Sn(OtBu)_4_ in BnNH_2_, whereby a gel was formed directly during the nanoparticle synthesis. The other strategy is based on synthesizing the ITO nanoparticles and arranging them into a macroscopic network in a subsequent step. The synthesis of the nanoparticles was realized by autoclave-assisted heating of In(acac)_3_ and Sn(OtBu)_4_ in BnOH and, alternatively, microwave-assisted heating of In(acac)_3_ and tin(IV) bis(acetylacetonate)dichloride in BnOH and oleylamine (OAm).

### 2.1. Nonaqueous One-Pot Synthesis of Aerogel **A**

**Figure 1:**
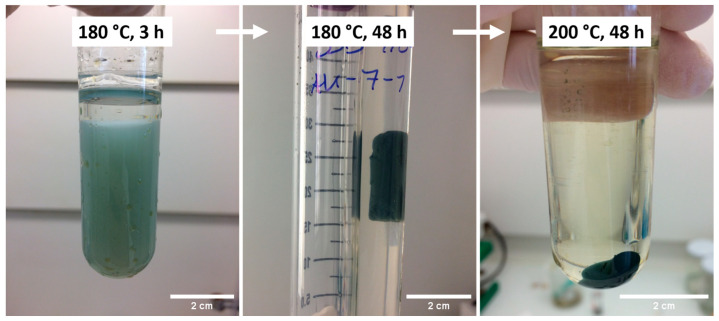
Photographs of the obtained synthesis products for different synthesis durations and temperatures in the nonaqueous one-pot sol–gel process.

The nonaqueous one-pot synthesis in BnNH_2_ resulted in a blue cylindrical ITO gel consisting of crystalline ITO nanoparticles. Figure 1 shows photographs of the obtained gels for different synthesis temperatures and reaction times. While after 3 h of synthesis, almost the whole reaction mixture converted into a greenish–blue gel with low structural stability, for longer synthesis durations, smaller gel monoliths with improved mechanical stability were observed. Higher synthesis temperatures resulted in further shrinkage of the gels.

**Figure 2:**
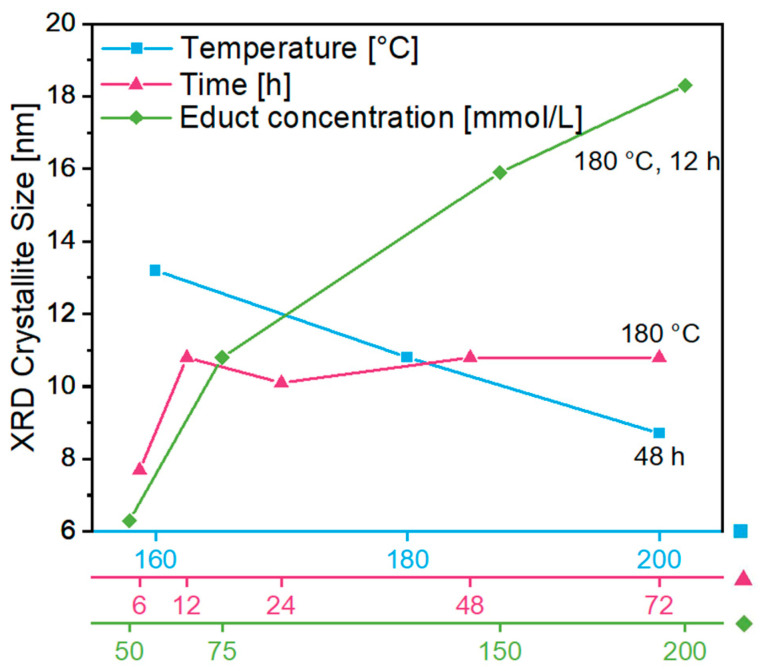
XRD crystallite size of the obtained gel products after drying to powders as a function of different synthesis parameters: effect of synthesis temperature at a reaction duration of 48 h (blue squares), influence of reaction duration at a synthesis temperature of 180 °C (rose triangles) and effect of the In(acac)_3_ concentration at 180 °C for 12 h (green diamonds).

To achieve a better understanding of the structural characteristics of the gel, in one series of investigations, the obtained products were directly dried after removal from the reaction medium and immersion in acetone and analyzed by XRD (the diffractograms can be found in the Supplementary Materials section, Figure S1). Thereby, the crystallinity and crystallite size of the ITO nanoparticles as the building blocks of the gels were determined. To understand the influence of the synthesis on the product properties, a variation of the synthesis parameters, in particular the temperature, time and educt concentration, was performed (Figure 2). The observed tendencies are presented in further detail in Tables S1–S3 in the Supplementary Materials section.

As tin is incorporated in the lattice only at relatively low doping levels, in the X-ray powder diffractograms, only the cubic bixbyite structure of In_2_O_3_ (ICDD PDF No. 01-088-2160) was detected, without the presence of any additional phase (Figure S1a). First, the effect of a variation of the synthesis temperature in the range of 160–200 °C with a synthesis duration of 48 h was monitored. The determined mean crystallite size of the washed and dried ITO products decreased from 13.2 to 8.7 nm (Figure 2: blue squares), as well as the gel volume (Figure 1). Moreover, the influence of a synthesis duration of 3 to 72 h at a temperature of 180 °C was investigated. The X-ray powder diffractograms of the dried ITO particles in Figure S1b show sharpening of all reflections as the synthesis duration increased from 3 to 12 h due to the growing crystals. This effect can be confirmed in Figure 2 (rose triangles), showing increasing crystallite sizes from 7.7 to 10.8 nm up to 12 h of synthesis, with no further growth with longer synthesis durations. After 3 h of synthesis, the whole volume of the reaction mixture converted into a blue gel with a low structural stability, whereas for higher synthesis durations, shrinking of the gel was observed, leading to improved stability (Figure 1). Finally, the concentration of the indium precursor was varied in the range of 25–200 mmol/L while keeping the reaction temperature constant at 180 °C for a reaction time of 12 h. At a concentration of 25 mmol/L, no gel was obtained, but a sort of light green gel-like intermediate with a high viscosity and slight transparency was obtained. Because of the amorphous nature of the particles according to the X-ray powder diffractogram (Figure S1c), no crystallite size could be calculated. The powder XRD patterns indicate an increasing crystallinity and a growth in crystallite size from 6.3 nm at a concentration of 50 mmol/L to 18.3 nm at 200 mmol/L indium precursor concentration (Figure 2: green diamonds).

These observations reveal that an educt concentration of at least 50 mmol/L at 180 °C and a synthesis duration of 12 h are required to form a gel during the reaction. Moreover, the gel volume decreases with increasing synthesis temperature, duration and educt concentration. In view of a future preparation at a larger scale, low synthesis temperatures, short durations and high yields are intended, aiming for lower costs and higher efficiency. According to the investigation, a compromise can be found with respect to the gel volume (which can be taken as a rough indication of yield), its stability and crystallinity and the crystallite size of the constituent ITO nanoparticles. Therefore, the following synthesis parameters were chosen as optimal for the efficient preparation of ITO gels in the one-pot process: 180 °C, 12 h and a concentration of 75 mmol/L of the indium precursor. After solvent exchange of the gel in acetone and CPD, a cylindrical monolithic aerogel was obtained, which is referred to as aerogel **A** in this study.

**Figure 3:**
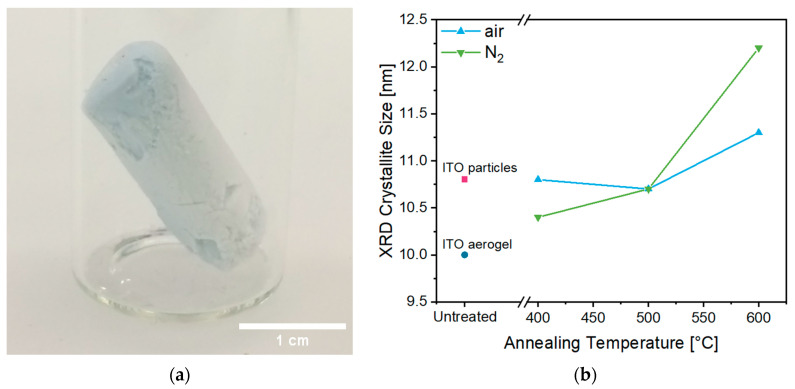
Photograph of an ITO aerogel **A** (15 at.% Sn) directly after CPD (**a**) and the calculated XRD crystallite size (**b**) of the washed and dried ITO product (rose square), the ITO aerogel (dark blue circle) after CPD and the calcined aerogels as a function of the annealing temperature in air (blue triangles) and N_2_ (green diamonds).

The cylindrical ITO aerogel **A** (Figure 3a) had a diameter of about 1 cm, a length of 2.7 cm and a density of about 0.1 g/cm^3^ calculated by weighing. Heat treatment led to further shrinkage and caused a color change from blue to yellow in air and to an increasing appearance of a grey color in a N_2_ atmosphere. According to Lee et al., the blue color of the product can be explained by in situ reduction conditions during the synthesis [33]. A blue color indicates the presence of oxygen vacancies, resulting in increasing electronic conductivity. Saturation of these vacancies through oxidation, e.g., by annealing in air, causes an increasing resistivity and a color change to yellow [33]. The greyish color after annealing in N_2_ might indicate a carbon layer resulting from organic residues adsorbed on the aerogel surface.

Phase purity was observed in the as-synthesized product after drying, as well as the critical point-dried aerogel and the heated aerogels, without noticeable changes in crystallinity, as observed for the peak patterns at higher heating temperatures (Figure S2a). Interestingly, the initial gel product already consisted of crystalline ITO nanoparticles, in contrast to previous reports of ITO aerogels in which annealing or calcination was still required for crystallization [19,34]. This is a great advantage, as the gel properties can be adjusted easily during the initial synthesis. The crystallite size (Figure 3b) for the as-synthesized product after drying (rose square) was calculated as 10.8 nm, and that for the aerogel after CPD (dark blue circle) was calculated as 10 nm, which might be due to the loss of some larger particles during the washing and CPD procedures, whereas annealing leads to larger crystallite sizes as a consequence of the sintering procedure of the particles in the range of 10.8 to 11.3 nm in air (blue triangles) and between 10.4 and 12.2 nm in a N_2_ atmosphere (green diamonds). Nevertheless, all observed size changes were small, indicating that the crystallinity essentially remained unchanged during all treatment steps after the synthesis. Notably, the determined crystallite sizes were smaller than those previously reported for an ITO aerogel, which was described as showing a crystallite size of about 20 nm after annealing at 600 °C in air [19].

**Figure 4:**
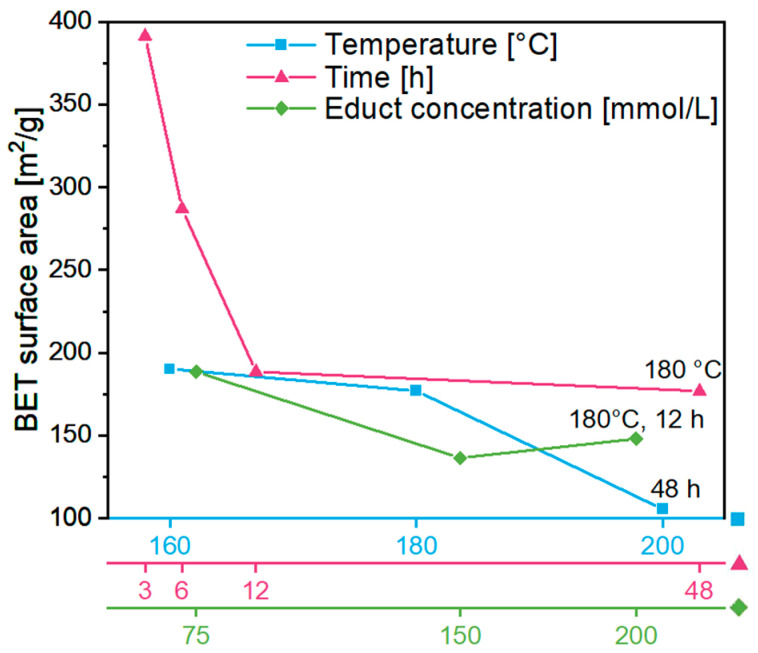
BET surface area as a function of different synthesis parameters. Effect of synthesis temperature at a duration of 48 h (blue squares), the effect of duration at a temperature of 180 °C (rose triangles) and the effect of In(acac)_3_ concentration at 180 °C for 12 h (green diamonds).

As shown above, the ITO gels obtained from the synthesis had considerably different volumes depending on the synthesis parameters. After conversion into ITO aerogels through solvent exchange and CPD, nitrogen sorption measurements were performed to determine their Brunauer–Emmett–Teller (BET) surface areas (Figure 4). As the synthesis temperature was increasing from 160 to 200 °C for 48 h, the gel volume decreased, and similarly, the BET surface area shrank from 190.1 to 105.4 m^2^/g, corresponding to a decrease in total pore volume from 0.8 to 0.3 cm^3^/g (Figure S6a). Similar to the variation of the synthesis temperature, a decreasing gel volume resulted in a decrease in BET surface area from 391.4 to 176.9 m^2^/g and a reduction in total pore volume from 1.7 to 0.7 cm^3^/g based on reaction durations of 3–48 h at 180 °C. As expected, at higher educt concentrations, the volume of the resulting gel (180 °C, 12 h) decreased, as did the BET surface area, showing values of 188.6 m^2^/g at 75 mmol/L and 148.1 m^2^/g at 200 mmol/L. Since the BET surface area and the total pore volume decreased with decreasing gel volume, a correlation between them was confirmed. The corresponding investigations of the annealed aerogels are discussed in Section 2.3.

**Figure 5:**
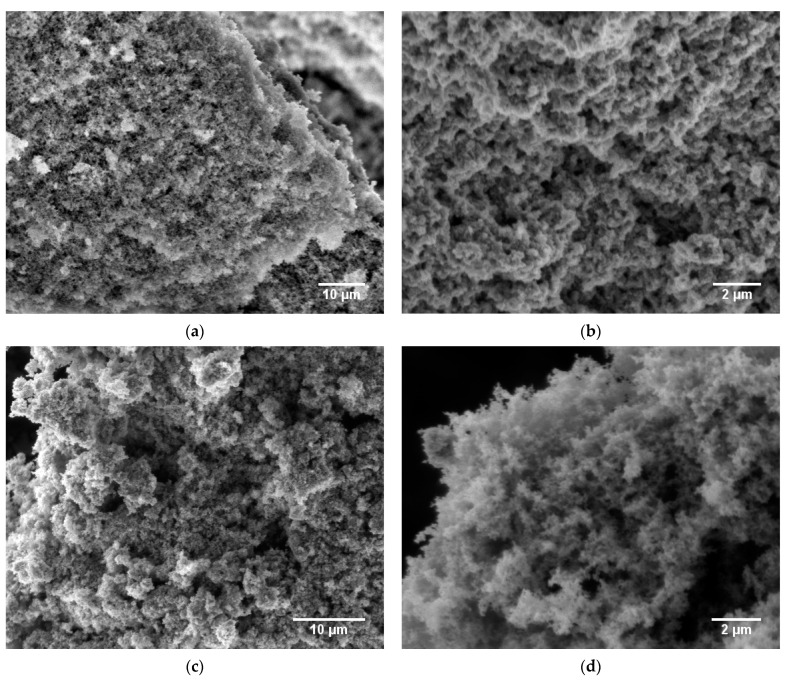
SEM micrographs of ITO aerogel **A** samples as obtained directly after CPD (**a**,**b**) and after annealing at 400 °C for 2 h in air (**c**,**d**) at different magnifications.

Figure 5 shows SEM micrographs of the untreated (Figure 5a,b) and annealed (Figure 5c,d) aerogel **A** samples at different magnifications, which point to an open, porous material. The images at low magnification (Figure 5a,c) indicate a mesoporous, fluffy microstructure comparable with images previously reported for ITO aerogels [19]. Comparison of the untreated and heated aerogels at high magnification indicates that the nanostructure was enhanced by the annealing treatment. According to the crystallite size calculated from XRD, annealing in air at 400 °C caused no crystallite growth, but it had an effect on the aerogel structure. Figure 5d reveals an aerogel with a finer nanostructure, which can be explained by the removal of surface organics. These findings are supported by TGA, as shown in Figure S8a, which proves an organic content of about 6.1 wt.% in the untreated aerogel. To the best of our knowledge, no TGA data for ITO aerogels have been reported to date. Elemental analysis via energy-dispersive X-ray spectroscopy (EDX) reveals only negligible changes in the carbon, hydrogen and nitrogen contents of the aerogels resulting from the annealing treatment.

**Figure 6:**
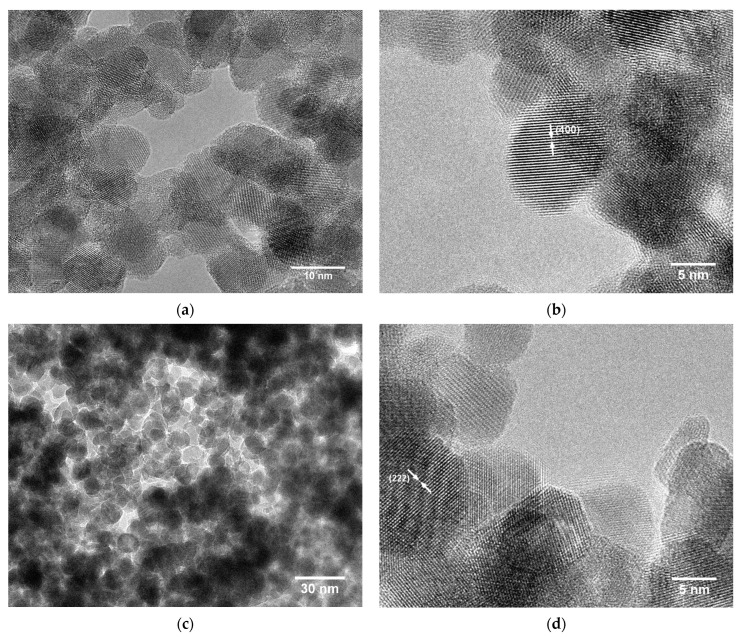
HRTEM micrographs of ITO aerogel **A** samples as obtained directly after CPD (**a**,**b**) and after annealing at 400 °C for 2 h in air (**c**,**d**) at different magnifications.

HRTEM images of the untreated aerogel (Figure 6a,b) and the aerogel after annealing at 400 °C (Figure 6c,d) show the presence of lattice fringes, indicating that the aerogels consist of highly crystalline particles. The well-defined particles are nearly spherical and show random orientation with respect to each other, as the visible lattice fringes could be assigned to different crystal planes (only one indexing per sample is shown for a better overview). The crystallinity and shape correspond well to the data on ITO nanoparticles from the nonaqueous synthesis in BnOH reported by Ba et al. [35]. Annealing causes particle aggregation due to particle sintering, which is shown in Figure 6c. Furthermore, the measured particle diameters in the range of 9.7–11.1 nm for the untreated and heated aerogels confirm the crystallite sizes as determined by XRD analysis.

For a better understanding of the assembly process of the nanoparticles into a gel during synthesis, the effect of the solvent was investigated. ITO nanoparticles were previously obtained in BnOH. In contrast, the use of BnNH_2_ as the solvent resulted in gel formation directly during synthesis. BnNH_2_ features a primary amine group, and due to the similarity to BnOH, we infer that this substituent and the fact that it leads to alkaline conditions is the cause for instant gelation. Consequently, an analogous synthesis was performed in a solvent consisting of a benzene unit with two primary amine substituents, m-XDA. The nanoparticles arranged in a gel, and XRD analysis of the product after drying proved the presence of a bixbyite phase with similar phase purity but clearly reduced crystallinity (Figure S2b), corresponding to a smaller average crystallite size of 6.2 nm. As m-XDA has two primary amine groups for coordination to the forming nanoparticles, it might interfere with the particle growth during the synthesis, resulting in a lower crystallinity, similar to the effect of multifunctional ligands reported in previous investigations [26,36].

An aerogel was obtained via solvent immersion and CPD, with similar appearance to that observed for aerogel **A**. However, compared to the BnNH_2_ system, the as-synthesized gel has a larger volume, as confirmed by gas sorption measurements of the aerogel, indicating a 3 times larger total pore volume (Figure S6c) and a 1.4 times higher BET surface area (Figure S5). Heat treatment in air at 400 °C for 2 h resulted in a color change from blue to yellow (Figure S9a) and peak sharpening of the XRD reflections, corresponding to an increase in crystallite size from 6.2 to 7.8 nm after heat treatment (Figure S4). In addition, TGA measurements (Figure S9b) showed an organic content of about 20 wt.% for the aerogel after CPD, which was almost completely removed after annealing. Thus, we can infer that the surface-adsorbed organics could cause large pore diameters, leading to the high volume of the gel.

### 2.2. Synthesis of Nanoparticles and Their Assembly into Aerogels **B** and **C**

**Figure 7:**
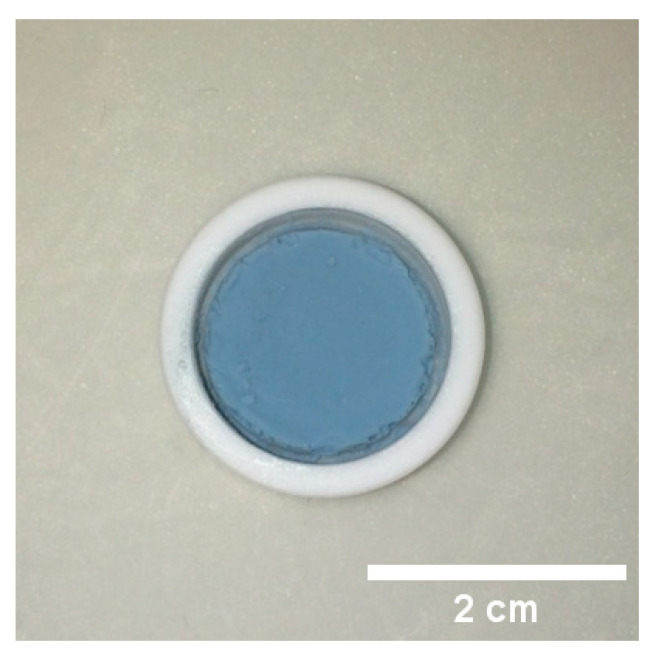
Photograph of ITO aerogel **B** (15 at.% Sn) after CPD in a Teflon cup.

In the following section, the alternative preparation strategy of ITO aerogels from preformed nanoparticles is discussed. The ITO nanoparticles were synthesized in BnOH via autoclave-assisted heating and, alternatively, a microwave-assisted heating step, then assembled into macroscopic aerogels through CPD. Both syntheses resulted in blue precipitates, which, after washing, were directly redispersed in a mixture of MEEAA and H_2_O. For other kinds of nanoparticles, a mixture of MEEAA and ethanol has typically been utilized, inducing destabilization by the addition of H_2_O [17,37,38]. In this case, a different approach was applied, since the nanoparticles showed the highest stability in H_2_O. Rapid and efficient coagulation of the ITO nanoparticle dispersion was achieved by the addition of two drops of 1 M NaCl; however, only heating at 70 °C for 30 min generated a thixotropic gel. When applying lower shear forces, the gel lost stability and liquefied again. Therefore, a very gentle solvent exchange procedure was performed, followed by CPD, resulting in blue, disc-shaped ITO aerogels (Figure 7). The obtained ITO aerogel from the autoclave-assisted route (200 °C, 48 h) was named aerogel **B**, and the product of the microwave-assisted route (220 °C, 10 min) is referred to as aerogel **C** [4,35]. Aerogel **B** shrank to 74.9 % of the original volume of the dispersion after CPD and had a density of about 0.1 g/cm^3^, whereas aerogel **C** had a similar appearance, shrinking to 65.1 % after CPD. In both cases, annealing in air led to a yellow aerogel, as was also observed for aerogel **A**.

**Figure 8:**
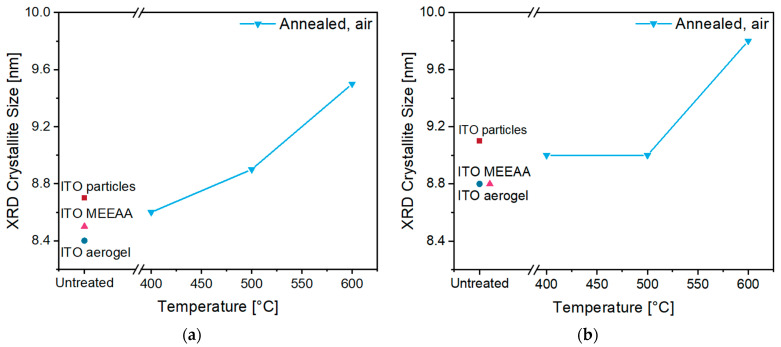
Crystallite size of ITO aerogels **B** (**a**) and **C** (**b**) in comparison to the respective as-synthesized ITO nanoparticles (red squares), MEEAA-functionalized nanoparticles (rose triangles), the ITO aerogels after CPD (dark blue circles) and the aerogel after annealing in air (blue downward triangles) at temperatures of 400–600 °C as determined by XRD.

The X-ray diffractograms of the products of both syntheses indicate high crystallinity of both the initially formed nanoparticles and the aerogels. In all samples, a cubic bixbyite structure of In_2_O_3_ (ICDD PDF No. 01-088-2160) was observed without crystalline impurities. Furthermore, no significant changes in the reflections during annealing at temperatures of 400–600 °C in air were observed (Figure S3). Nevertheless, the crystallite size of the aerogels remained practically constant up to 400–500 °C but increased significantly during calcination at 600 °C (Figure 8).

**Figure 9:**
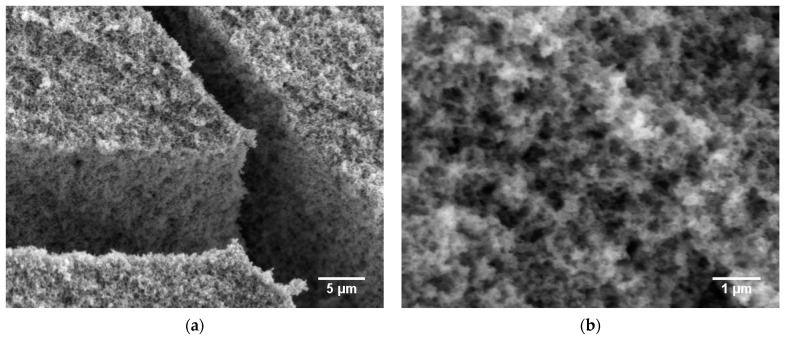

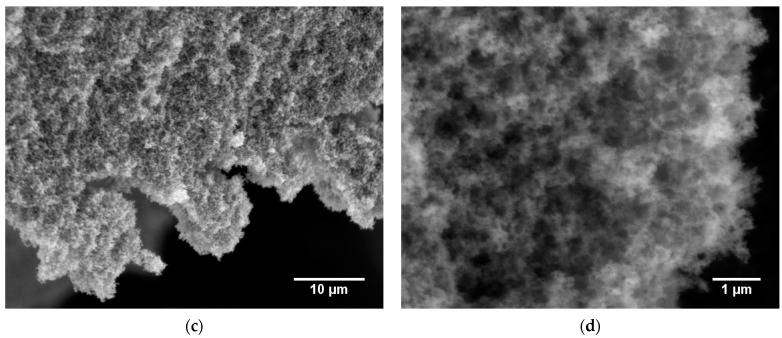
SEM micrographs of the ITO aerogel **B** samples as obtained directly after CPD (**a**,**b**) and after annealing at 400 °C for 2 h in air (**c**,**d**) at different magnifications.

The surface morphology of aerogel **B** directly after CPD and after annealing at 400 °C in air was analyzed using SEM (Figure 9). At low magnification, the surface morphology shows no significant differences between the two samples; the images confirm the high porosity of the obtained materials. In particular, Figure 9a provides an inside view through a crack, indicating that the whole material is highly porous. Comparison with aerogel **A** (Figure 5) points to a surface morphology with a higher homogeneity, and at higher magnification, the untreated aerogel (Figure 8b) exhibits a finer nanostructure due to smaller primary particles and a lower organic content of only 5.1 % (Figure S8b). This property was lost after annealing because of increasing crystallite sizes as a result of particle sintering.

**Figure 10:**
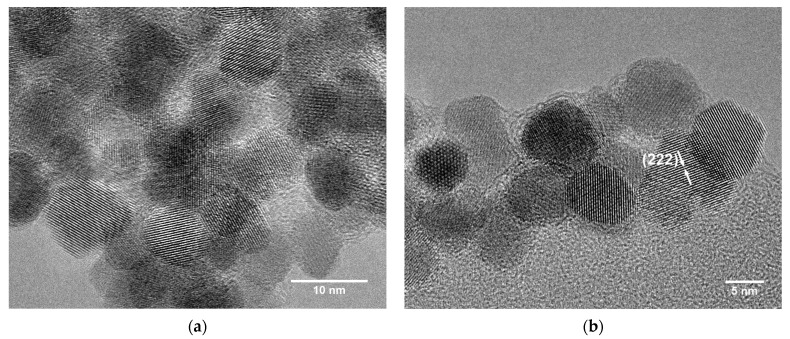

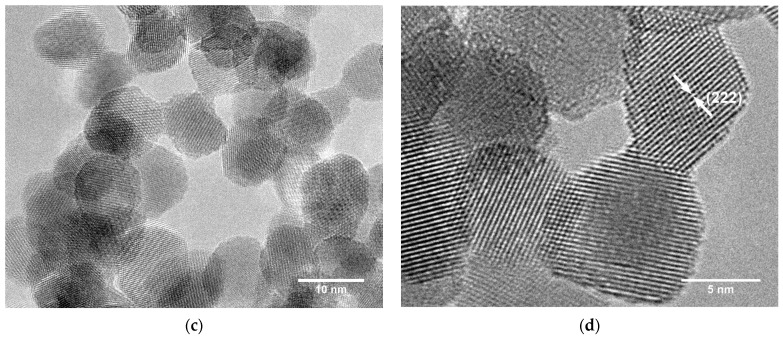
HRTEM micrographs of the primary nanoparticles in ITO aerogel **B** samples as obtained directly after CPD (**a**,**b**) and after annealing at 400 °C for 2 h in air (**c**,**d**) at different magnifications.

The HRTEM images presented in Figure 10 depict the interconnected nanoparticle network within the aerogels in further detail. The presence of crystalline particles is again evidenced by the visible lattice fringes. The nearly spherical particles are randomly connected, which points to the absence of oriented attachment. With measured particle diameters of about 9 nm, the XRD values are confirmed. After annealing, neck formation between neighboring particles due to sintering is clearly observed.

The gelation process was analyzed by investigating the arrangement of the ITO particles in a gel. Therefore, the functionalization of the particles with MEEAA and the surface-adsorbed organics were further investigated via ATR-IR spectroscopy, EDX spectroscopy and TGA. Due to the strong IR absorption properties of ITO, the identification of surface-adsorbed organics via ATR-IR spectroscopy was not possible (cf. Figure S7 and the discussion in the Supplementary Materials section). The organic content was compared between the functionalized particles and aerogels **B** and **C** via TGA measurements (Figure S8b,c) and EDX spectroscopy. In aerogel **B**, an organic content of 5.1 wt.% was found, which is a considerable drop compared to the functionalized nanoparticles, which showed an organic content of 10.6 wt.%. In the formation process of aerogel **C**, only a slight decrease in the amount of the surface-adsorbed organics from 9.7 to 7.4 wt.% was detected. Hence, the MEEAA content of the aerogels was greatly reduced during CPD. This was observed in an analogous fashion for BaTiO_3_ nanoparticles and their aerogels by Rechberger et al. and was therefore expected [17,38]. In combination with a high particle concentration, this effect leads to coagulation through attachment of the individual nanoparticles, which results in a gel. According to EDX spectroscopy, the samples contained no sodium or chloride after solvent exchange and CPD. Furthermore, we investigated which type of ions led to the destabilization of the dispersion. Therefore, ITO dispersions of 1 mL were destabilized with an aqueous KCl solution or a Na_2_SO_4_ solution, and each was heated at 70 °C for 30 min. The addition of KCl resulted in sedimentation of the particles, whereas after Na_2_SO_4_ addition, a thixotropic gel was obtained. These findings imply that sodium ions cause the coagulation of the nanoparticles, probably by weakening the coordination of MEEAA to the nanoparticle surface.

### 2.3. Investigation of Porosity and Conductivity of the Aerogels

**Figure 11:**
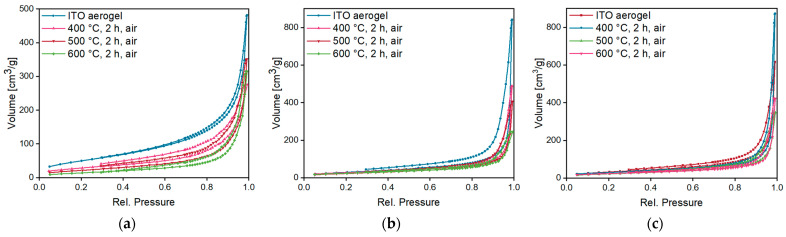
Nitrogen sorption isotherms of ITO aerogels **A** (**a**), **B** (**b**) and **C** (**c**) before and after annealing at temperatures of 400–600 °C in air (see Figure S11 for the corresponding BET plots).

Nitrogen sorption measurements were performed to compare the pore structure and the specific surface area of all three obtained ITO aerogels (Figure 11). In all cases, type III isotherms were obtained. Since adsorption increases significantly when applying high pressures, the isotherms indicate a macroporous adsorbent with weak interactions with the adsorptive [39,40,41]. The corresponding BET plots and their linear fits are presented in Figure S11 in the Supplementary Materials section. In comparison, Davis et al. observed type IV isotherms for their obtained ITO aerogels, indicating a mesoporous nature [19].

**Figure 12:**
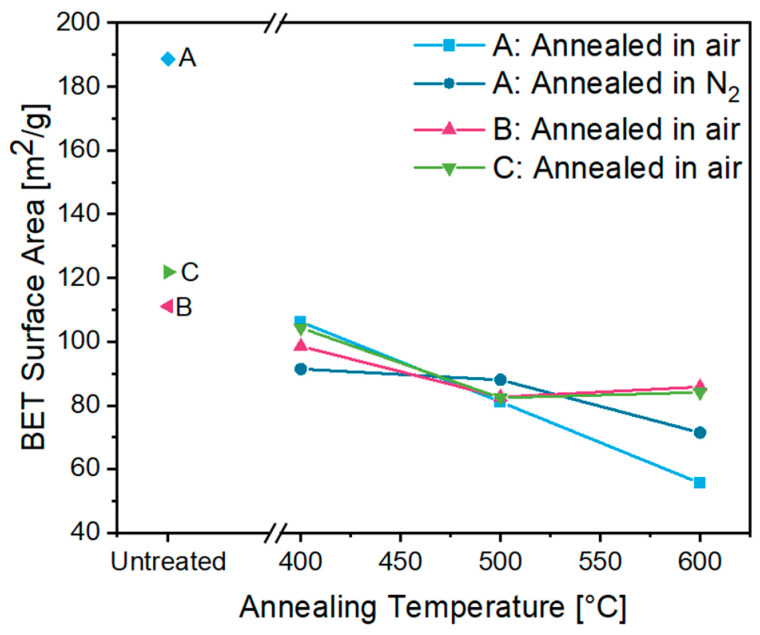
BET surface area of ITO aerogels **A** (blue diamond), **B** (rose left triangle) and **C** (green right triangle), and of the corresponding calcined aerogels after annealing in air or N_2_ at temperatures from 400 to 600 °C.

The BET surface area of the untreated aerogel **A** amounts to 188.6 m^2^/g, which is remarkably higher than for aerogels **B** and **C**, which showed values of 111.1 m^2^/g and 121.8 m^2^/g, respectively (Figure 12; see Figure S6b for total pore volume). This changes with the annealing procedure. After heat treatment, all aerogels, independent of the annealing atmosphere, had a rather similar surface area. With increasing annealing temperature, a decreasing surface area was obtained, with the largest decrease from 106.2 down to 55.6 m^2^/g observed for aerogel **A**. This effect was caused by Ostwald ripening and densification of the nanoparticles due to particle sintering [42]. The larger surface area in aerogel **A** supports the assumption mentioned above that the coordination of amine groups on the gel surface influences its porosity. Heat treatment leads to the removal of these groups, which apparently promotes nanoparticle sintering. The obtained values are close to previously reported BET surface areas of 310 m^2^/g and 117 m^2^/g for untreated ITO aerogels and 39 m^2^/g for aerogels annealed at 600 °C [19,34].

Figure S10 indicates a broad pore size distribution of the aerogels, with aerogel **B** showing a higher dependence on the annealing temperature compared to the other two aerogels. In this measurement, only pores smaller than 75 nm were analyzed because larger pores were inaccessible with N_2_ gas adsorption. Based on the SEM results discussed above, it is clear that large macropores are also present in the aerogels [43]. Mercury porosimetry is one possible way to analyze the sizes of macropores; however, the obtained materials were too fragile for such measurements.

**Figure 13:**
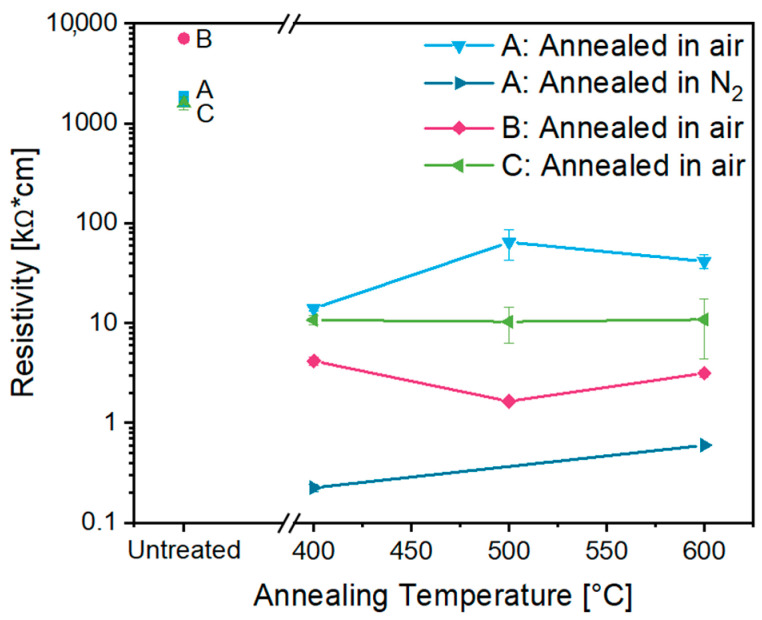
Electrical resistivity as a function of ITO aerogels **A** (blue square), **B** (rose circle) and **C** (green diamond) after CPD and after annealing in air and N_2_ at temperatures in the range of 400–600 °C.

The electrical conductivity of the obtained aerogels was analyzed by measuring their electrical resistivity via the four-point probe method. The electrical resistivity of the aerogels after CPD was higher than 1000 kΩ·cm, but annealing in air led to a considerable drop to 64.5–1.6 kΩ·cm (Figure 13). The highest annealing dependence was observed for aerogel **B**, with the electrical conductivity improved by almost three orders of magnitude. Although aerogel **C** had a lower doping concentration (10 at.% Sn), its electrical resistivity was in the same range as that of the other two aerogels. Annealing of aerogel **A** in a N_2_ atmosphere resulted in significantly lower values in the range of 0.2–0.6 kΩ·cm. This could be explained by the maintenance of oxygen vacancies in the ITO particles, which is known to result in an increased electrical conductivity, while oxidative conditions saturate oxygen vacancies, causing a lower conductivity [33]. Interestingly, an increase in annealing temperature hardly led to any changes in resistivity, whereas previous studies indicated a constant increase in conductivity [22,23]. According to Davis et al. and Rechberger et al., the electrical conductivity can be optimized by increasing the crystallinity and interparticle connection, varying the doping ratio and changing the pore structure [19,22]. Since the aerogels already showed high crystallinity even before the annealing treatment (Figures S2a and S3) and the optimum reported doping ratio of Sn was used, no further optimization of these parameters appears feasible [4,35]. Annealing seems to improve the particle–particle connections, leading to improved electrical conductivity, but once the connection is properly established, the conductivity values remain constant. Thus, we infer that the electrical conductivity might be further improved by varying the intrinsic properties of the nanoparticles. A similar nanoparticle-based conducting aerogel was recently reported by Rechberger et al. based on antimony-doped tin oxide (ATO) [22]. In this case, annealing in air at 400 °C led to a decrease in electrical resistivity by almost five orders of magnitude (from 100,000 kΩ·cm to 2 kΩ·cm), but increasing annealing temperatures up to 600 °C caused a further improvement, with a resistivity of only 0.08 kΩ·cm due to better connections between the particles. The lowest resistivity of 4.5 Ω·cm was observed for the ATO aerogel with a doping content of only 5 at.% Sb and a crystallite size of about 9 nm after annealing at 650 °C in air. According to Davis et al., who synthesized an ITO aerogel via an aqueous gelation process, after annealing at 600 °C in air, an electrical resistivity of 0.05 kΩ·cm was achieved [19]. The lower value can be explained by the direct gel formation from molecular precursors rather than the coagulation of nanoparticles with organic ligands. For non-porous ITO thin films with a thickness of 146 nm, Lee et al. reported an electrical resistivity of 5.2 mΩ·cm after annealing at 300 °C for 6 h in Ar and 5 % H_2_ [44]. This shows that in principle, ITO nanostructures can reach high electrical conductivity, but it needs to be taken into account that nanoparticle-based porous structures inevitably show much higher resistivity due to the contact resistance between the nanoparticles. Nevertheless, this comparison proves that the obtained ITO aerogels (**A**, **B** and **C**) show promising results, although further conductivity improvement is necessary to render the aerogels attractive for diverse applications.

## 3. Conclusions

Herein, we reported the successful assembly of conductive ITO nanoparticles into macroscopic aerogels via two different synthesis strategies using CPD and compared the properties of the resulting products in detail.

In one strategy, ITO aerogels were synthesized via a nonaqueous one-pot sol–gel method using BnNH_2_ as a solvent. During the synthesis in the autoclave, the formed nanoparticles were directly arranged into a gel, with the advantage that an additional gelation process could be omitted. In order to establish an efficient synthesis procedure, different parameters (temperature, duration and educt concentration of the indium precursor) were varied, and optimized parameters led to an ITO aerogel with high crystallinity and a BET surface area of 188.6 m^2^/g after solvent exchange and CPD. When replacing BnNH_2_ with m-XDA as the solvent, again a gel was obtained directly from the synthesis, leading to the conclusion that the amine substituents in the solvent are involved in the direct gelation process during the synthesis.

The other strategy involved the synthesis of ITO nanoparticles via nonaqueous sol–gel synthesis with BnOH as solvent through two different heating procedures, followed by their stabilization and processing into aerogels. To this end, the nanoparticles were first functionalized with MEEAA in an aqueous solution, and controlled destabilization was induced by the addition of NaCl. Subsequent solvent exchange and CPD resulted in disc-shaped blue aerogels.

The obtained ITO aerogels were compared in terms of several characteristics, in particular their porosity and electrical conductivity. While ITO aerogels reported in previous studies were obtained in amorphous form, in this study, highly crystalline primary nanoparticles were already present in the initially obtained aerogels. Moreover, all aerogels possessed a comparably large specific surface area, with a homogeneous porosity of the entire sample. Annealing at 400 °C in air resulted in a drop in electrical resistivity of about three orders of magnitude, while no significant further improvement was observed at higher annealing temperatures. The ITO aerogel obtained from the nanoparticles of the autoclave-assisted route showed the most promising result with the lowest electrical resistance of 1.6 kΩ·cm after annealing at 500 °C for 2 h in air, while after annealing in a nitrogen atmosphere, even lower values of 0.2 kΩ·cm were found for the one-pot synthesis. Our study shows that both one-pot synthesis strategies and the two-step assembly approach are viable for the preparation of nanoparticle-based aerogels with interesting properties. However, organics and, after calcination, the presence of grain boundaries are severe causes of high resistivity that still need to be addressed and improved to make these systems competitive with other approaches. The ITO aerogels obtained here show particular advantages due to their high crystallinity, even before calcination, and were obtained through experimentally simple and scalable processes.

## 4. Materials and Methods

### 4.1. Materials

Indium(III) acetylacetonate (In(acac)_3_, ≥99.99% metal basis), tin(IV) tert-butoxide (Sn(OtBu)_4_, ≥99.99% metal basis), benzylamine (BnNH_2_, for GC derivatization, ≥99%), tin(IV) bis(acetylacetonate)dichloride (98%), benzyl alcohol (BnOH, anhydrous, 99.8%), m-xylylenediamine (m-XDA, ≥99%), oleylamine (OAm, technical grade, 70%), 2-[2-(2-methoxyethoxy)ethoxy]acetic acid (MEEAA, technical grade), tetrahydrofuran (≥99.9%), sodium chloride (>99% ACS reagent), potassium chloride (>99.99% trace metals basis) and sodium sulfate (≥99.99% trace metals basis) were purchased from Merck KGaA (Darmstadt, Germany), and acetone (absolute) was purchased from Fisher Scientific (Waltham, MA, USA). All chemicals were used without further purification. Liquid carbon dioxide (CO_2_) and nitrogen (N_2_) were provided by PanGas AG (Dagmersellen, Switzerland).

### 4.2. Nonaqueous One-Pot Sol–Gel Synthesis

#### Synthesis of ITO Aerogel

The tin-doped (Sn/(Sn + In) = 15 at.%) indium oxide gel was synthesized using autoclave-assisted nonaqueous one-pot synthesis with BnNH_2_ as a reaction medium. The reaction mixture was prepared in a humidity- and oxygen-free glovebox under an argon atmosphere. A total of 1.5 mmol In(acac)_3_ (618.2 mg) and 0.26 mmol Sn(OtBu)_4_ (108.8 mg) were dissolved in a vial with 20 mL BnNH_2_. The slightly yellowish reaction mixture was transferred into a Teflon liner, which was placed into a stainless steel autoclave that was sealed and heated in a furnace (Memmert UFE 400, Memmert, Schwabach, Germany) at 180 °C for 12 h. The obtained blue gel was immersed three times for 24 h in pure acetone for liquid exchange within the pores, followed by supercritical drying in CO_2_ with a SPI-DRY Critical Point Dryer 13200 (Structure Probe, Inc., West Chester, PA, USA). The result was a blue cylindrical nanoparticle-based aerogel.

### 4.3. Nonaqueous Sol–Gel Synthesis of the Nanoparticles and Their Assembly into Aerogels

#### 4.3.1. Autoclave Synthesis of ITO Nanoparticles

The tin-doped (Sn/(Sn + In) = 15 at.%) indium oxide nanoparticles were synthesized analogously, replacing BnNH_2_ with BnOH, following the protocol described in Ref. [35]. The reaction was carried out at 200 °C for 48 h. The resulting ITO nanoparticles were separated by centrifugation, obtaining a blue precipitate, which was washed three times in acetone.

#### 4.3.2. Microwave Synthesis of ITO Nanoparticles

For comparison with the product of another type of synthesis, tin-doped (Sn/(Sn + In) = 10 at.%) indium oxide nanoparticles were synthesized using a microwave-assisted route. The synthesis was carried out following the protocol described by Müller et al. [4] for the preparation of antimony-doped tin oxide particles. The reaction mixture was again prepared in a glovebox under an argon atmosphere, dissolving 0.45 mmol In(acac)_3_ (185.5 mg) and 0.05 mmol tin(IV) bis(acetylacetonate)dichloride (19.4 mg) in a vial with a mixture of 2.5 mL BnOH and 2.5 mL OAm, resulting in a slightly yellowish solution. The vial was sealed with a Teflon cap and heated outside the glovebox in a microwave reactor (CEM Discover, 2.45 GHz, CEM GmbH, Kamp-Lintfort, Germany) at 220 °C for 10 min with a high stirring rate. The resulting blue precipitate was separated by centrifugation and washed three times in acetone.

#### 4.3.3. ITO Dispersion, Gelling and Supercritical Drying

For the gelation procedure, 200 mg of the separated nanoparticles (from the autoclave or microwave synthesis) was dispersed in 5 mL of a 0.3 M MEEAA solution in deionized water by stirring for 3 h at room temperature. In order to remove the residual and unbound MEEAA, tetrahydrofuran (THF) was added in a volumetric ratio of at least 5:1, leading to agglomeration of the nanoparticles. The functionalized particles were extracted by centrifugation, redispersed in 1 mL deionized water and transferred into a Teflon cup. To induce destabilization, 2 drops of a 1 M NaCl solution in deionized water were added, and the dispersion was heated in a saturated water atmosphere at 70 °C for 30 min in an oven (Binder GmbH, Tuttlingen, Germany). Afterwards, the obtained blue thixotropic gel was immersed three times for 24 h in pure acetone for liquid exchange from the pores, followed by supercritical drying in CO_2_ with a SPI-DRY Critical Point Dryer 13200 (Structure Probe, Inc., West Chester, PA, USA). The result was a blue disc-shaped nanoparticle-based aerogel.

### 4.4. Heat Treatment

The aerogels were placed inside covered alumina-based ceramic crucibles and annealed for 2 h in air (Nabertherm P 330 furnace, Nabertherm GmbH, Lilienthal, Germany) under an N_2_ atmosphere (Carbolite-EHA-Compact tube furnace, Carbolite Gero GmbH & Co. KG, Neuhausen, Germany) at 400–600 °C employing a heating rate of 1 K/min, followed by slow cooling to room temperature inside the furnace.

### 4.5. Characterization

X-ray powder diffraction (XRD) was measured on a PANalytical Empyrean device (Malvern PANalytical, Malvern, UK) equipped with a PIXcel 1D detector (Malvern PANalytical, Malvern, UK) using Cu Kα radiation. The average crystallite size (L) was calculated on the (222) peak broadening via the Scherrer equation.

After outgassing the samples at 100 °C for 24 h on a Quantachrome Autosorb iQ (Anton Paar AG, Graz, Austria), their surface area was analyzed by N_2_ gas sorption measurements at 77 K. In addition to determination of the surface area via the Brunauer–Emmett–Teller (BET) method, the pore size and the pore volume were determined by density functional theory (DFT) with a nonlocal DFT calculation model based on silica cylindrical pores [45].

Thermogravimetric analysis (TGA) was performed using a TGA Mettler Toledo SDTA851e system (Mettler-Toledo GmbH, Greifensee, Switzerland) with a heating rate of 5 K/min in air in the range of 25–1000 °C. Carbon elemental analysis was performed on a device from LECO (LECO Corporation, St. Joseph, MI, USA).

The destabilizing effect of the Na^+^ and Cl^–^ ions was examined using two dispersions (1 mL each) with a particle concentration of at least 200 mg/mL. In one dispersion, 6 drops of a 1 M KCl solution in deionized water and in the other one 4 drops of a 0.5 M Na_2_SO_4_ solution in deionized water were added, and the mixtures were heated at 70 °C for 30 min.

Prior to microstructural investigation, the scanning electron microscope (SEM) samples were placed on an aluminum sample holder and fixed with a silver paste. Microscopy and energy-dispersive X-ray (EDX) spectroscopy were carried out on a Leo 1530 SEM Gemini system (Carl Zeiss AG, Oberkochen, Germany). EDX analysis was performed at three different locations with a life time of 300 s. High-resolution transmission electron microscopy (HRTEM) was performed on an FEI Talos F200X device (Thermo Fisher Scientific Inc., Waltham, MA, USA) operated at 200 kV after the samples were dispersed in ethanol, transferred onto a carbon copper grid on filter paper and dried in air at room temperature.

For the resistivity measurements, a Keithley 237 high-voltage source measurement unit (SMU, Tektronix Inc., Beaverton, OR, USA) with a cylindrical 4-point probe head from Jandel (spacing s = 1 mm, Jandel Engineering Ltd., Leighton Buzzard, UK) was used. The probes were placed on the sample with a weight of 1.6 to 5.1 g, as measured on a simple gravimetric scale. Through the two outer probes, a current in the range of 0.01–100 μA was applied, whereas the voltage was measured with the two inner probes. Finally, the electrical resistivity was calculated according to Ohm’s law multiplied by a geometric correction factor of G = 2π × s × 0.951 = 0.6 cm by choosing the approximation for an infinite plane of a thickness of t = 2.5 mm [46].

## Data Availability

The data presented in this study are available upon request from the authors.

## Supplementary Materials

The following supporting information can be downloaded at: https://www.mdpi.com/article/10.3390/gels9040272/s1, Table S1: Tendency of different characteristics at a duration of 48 h by increasing synthesis temperatures, at a temperature of 180 °C by increasing synthesis durations and after annealing of the aerogel for 2 h by increasing annealing temperatures; Table S2: Tendency of different characteristics at 180 °C for 12 h by increasing educt concentrations; Table S3: Tendency of different characteristics at 180 °C for 12 h by replacing BnNH_2_ with m-XDA; Figure S1: X-ray diffractograms showing the effect of different synthesis parameters on the crystallinity of the obtained product from the nonaqueous one-pot sol–gel process: synthesis temperature (a), duration (b) and educt concentration (c); Figure S2: X-ray diffractograms of the products of the nonaqueous one-pot sol–gel process in BnNH2: the as-synthesized ITO product after drying (blue), ITO aerogel after CPD (dark blue) and the aerogels after annealing in air in the temperature range of 400–600 °C (a) and their comparison with the aerogel obtained with m-XDA before and after annealing (b); Figure S3: X-ray diffractograms of the as-synthesized ITO nanoparticles (blue), ITO aerogel after CPD (dark blue) and the aerogels after annealing in air in the temperature range of 400–600 °C using approaches **B** (a) and **C** (b); Figure S4: Crystallite size of the ITO aerogels as obtained in BnNH_2_ and m-XDA and after annealing at 400 °C in air; Figure S5: Comparison of the BET surface area of the ITO aerogels after CPD (rose squares) and annealed aerogels in air at 400 °C (blue circles) from the nonaqueous one-pot sol–gel process in BnNH_2_ and m-XDA; Figure S6: Total pore volume as a function of different synthesis temperatures for a duration of 48 h (blue squares), as a function of duration at a temperature of 180 °C (rose triangles) and as a function of the educt concentration of the indium precursor at 180 °C for 12 h (green diamonds) (a); total pore volume as a function of ITO aerogel **A** after CPD (blue diamond), **B** (rose left triangle) and **C** (green right triangle) and after annealing in air in the temperature range of 400–600 °C (b); comparison of the total pore volume from the ITO aerogels before and after annealing at 400 °C in air in the nonaqueous one-pot sol–gel process (c); Figure S7: ATR-IR measurements of MEEAA (green); a 1 M NaCl solution (blue); and a solution containing 0.3 M MEEAA, H_2_O and 1 M NaCl (rose); Figure S8: TGA results of aerogel systems A (a), B (b) and C (c) comparing the respectively obtained ITO particles after direct drying of the as-synthesized product (rose), the ITO nanoparticles after stabilization with MEEAA for systems B and C (green) and the respective ITO aerogels obtained after CPD (blue); Figure S9: ITO aerogel (15 at.% Sn) obtained via the nonaqueous one-pot sol–gel process in m-XDA before (blue) and after annealing at 400 °C in air (yellow) in glass vials (a) and TGA results (b) of the aerogel after CPD (blue) and annealing at 400 °C in air (rose); Figure S10: Pore size distribution (from 2 to 75 nm) by DFT analysis of N_2_ gas sorption measurements of ITO aerogels **A** (a), **B** (b) and **C** (c) showing the aerogels directly after CPD (blue) and annealed aerogels at 400 °C (rose), 500 °C (red) and 600 °C (green) in air; Figure S11: BET plots derived from the corresponding nitrogen sorption isotherms of ITO aerogels A (a), B (b) and C (c) presented in Figure 11. Good linear fits were obtained in all cases.
