# Supplementary material for: Conducting ITO Nanoparticle-Based Aerogels—Nonaqueous One-Pot Synthesis vs. Particle Assembly Routes
Source: Gels. 2023 Mar 25;9(4):272. doi: 10.3390/gels9040272 (PMC10138307; doi:10.3390/gels9040272)
Supplement: Supplementary file 1 [file gels-09-00272-s001.zip › gels-2273744-supplementary.pdf]

gels

MDPI

Article

Conducting ITO Nanoparticle-Based Aerogels—Nonaqueous One-Pot Synthesis vs. Particle Assembly Routes

Samira Sang Bastian [1], Felix Rechberger [2], Sabrina Zellmer [1,3], Markus Niederberger [2] and Georg Garnweitner [1,4,*]

[1] Institute for Particle Technology, Technische Universität Braunschweig, Volkmaroder Str. 5, 38104 Braunschweig, Germany
[2] Laboratory for Multifunctional Materials, Department of Materials, ETH Zurich, Vladimir-Prelog-Weg 5, 8093 Zurich, Switzerland
[3] Fraunhofer Institute for Surface Engineering and Thin Films (IST), Bienroder Weg 54E, 38108 Braunschweig, Germany
[4] Laboratory for Emerging Nanometrology, Technische Universität Braunschweig, Langer Kamp 6A, 38106 Braunschweig, Germany
* Correspondence: g.garnweitner@tu-braunschweig.de; Tel.: +49-5313919615

Table of Content:

1. Nonaqueous one-pot synthesis in $BnNH_2$
Tables S1–S3
2. X-ray diffractograms
2.1. Nonaqueous one-pot synthesis
Figures S1–S2
2.2. Nonaqueous sol-gel synthesis
Figure S3
3. XRD crystallite size
Figure S4
4. BET surface area by N_2 gas sorption measurement
Figure S5
5. Total pore volume by N_2 gas sorption measurement
Figure S6
6. ATR-IR spectroscopy
Figure S7
7. TGA measurements
Figure S8
8. ITO aerogel with m-XDA
Figure S9
9. Pore size distribution by DFT analysis of N_2 gas sorption measurements
Figure S10
10. BET plots derived from the isotherms in the N2 gas sorption measurements
Figure S11

1. Nonaqueous one-pot synthesis in $BnNH_2$

Table S1. Tendency of different characteristics at a duration of 48 h by increasing synthesis temperatures, at a temperature of 180 °C by increasing synthesis durations and after annealing of the aerogel for 2 h by increasing annealing temperatures.

Time	Temperature	Crystallinity	Crystallite size	Surface area	Total pore volume	Gel volume
48 h	160-210 °C	-	↓	↓	↓	↓
3-72 h	180 °C	↑	↑	↓	↓	↓
2 h	400-600 °C	-	↑	↓	-	-

Table S2. Tendency of different characteristics at 180 °C for 12 h by increasing educt concentrations.

Indium precursor	Crystallinity	Crystallite size	Surface area	Total pore volume	Gel volume
25 – 200 mmol/L	↑	↑	-	-	↓

Table S3. Tendency of different characteristics at 180 °C for 12 h by replacing $BnNH_2$ by m-XDA.

Medium	Crystallinity	Crystallite size	Surface area	Total pore volume	Gel volume	TGA
$BnNH_2$ – m-XDA	↓	↓	↑	↑	↑	↑

2. X-ray diffractograms

2.1. Nonaqueous one-pot synthesis

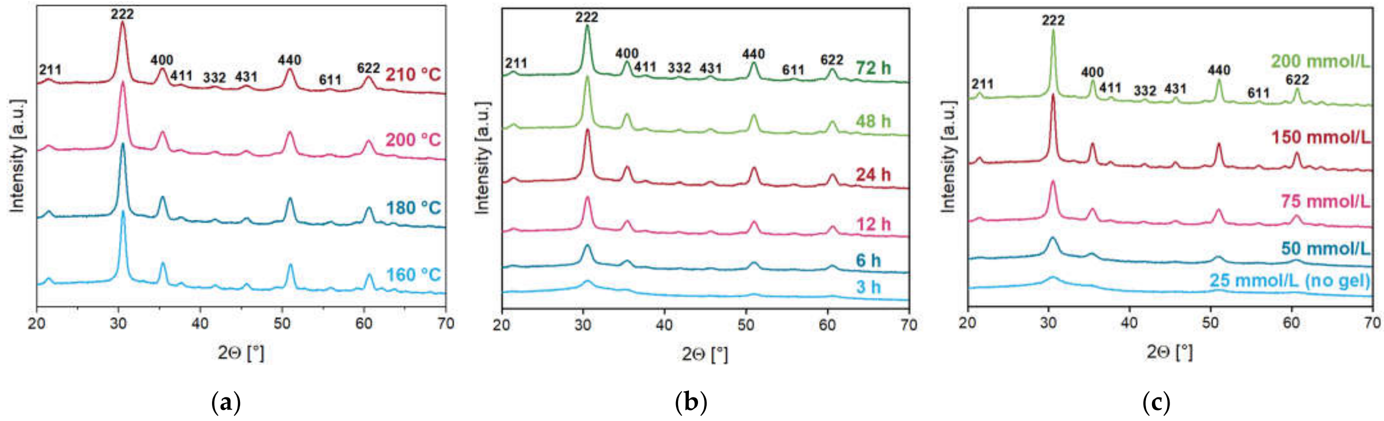

Figure S1. X-ray diffractograms showing the effect of different synthesis parameters on the crystallinity of the obtained product from the nonaqueous one-pot sol-gel process in $BnNH_2$. Synthesis temperature (a), duration (b), educt concentration (c).

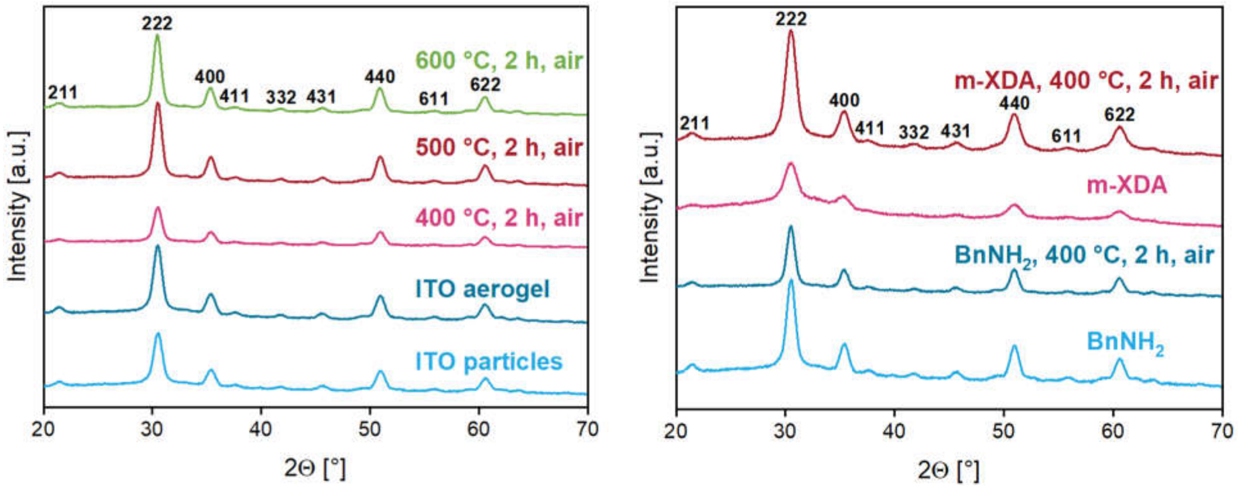

(**a**) (**b**)

Figure S2. X-ray diffractograms of the products of the nonaqueous one-pot sol-gel process in $BnNH_2$: the as-synthesized ITO product after drying (blue), ITO aerogel after CPD (dark blue) and the aerogels after annealing in air from 400 – 600 °C (a) and their comparison to the aerogel obtained with m-XDA before and after annealing (b).

2.2. Nonaqueous sol-gel synthesis

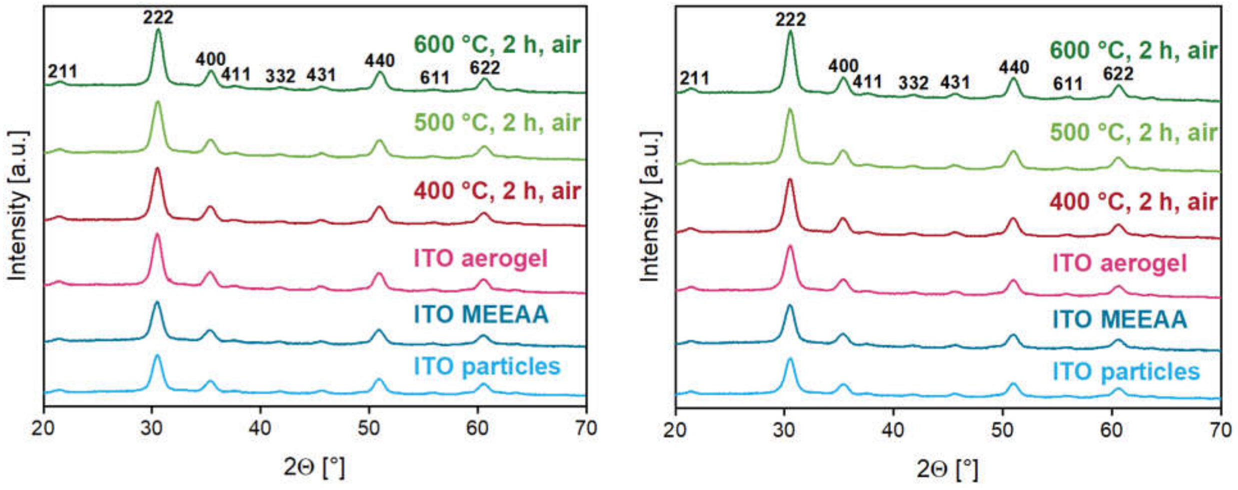

(**a**) (**b**)

Figure S3. X-ray diffractograms of the as-synthesized ITO nanoparticles (blue), ITO aerogel after CPD (dark blue) and the aerogels after annealing in air from 400 – 600 °C from approach **B** (a) and **C** (b).

3. XRD crystallite size

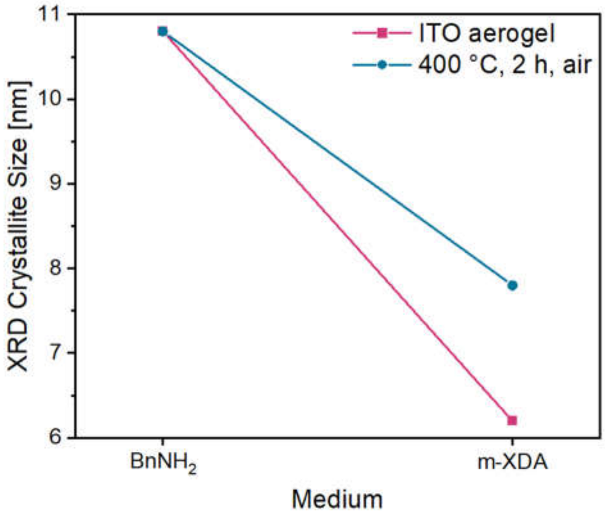

Figure S4. Crystallite size of the ITO aerogels as obtained in $BnNH_2$ and m-XDA, and after annealing at 400 °C in air.

4. BET surface area by N_2 gas sorption measurement

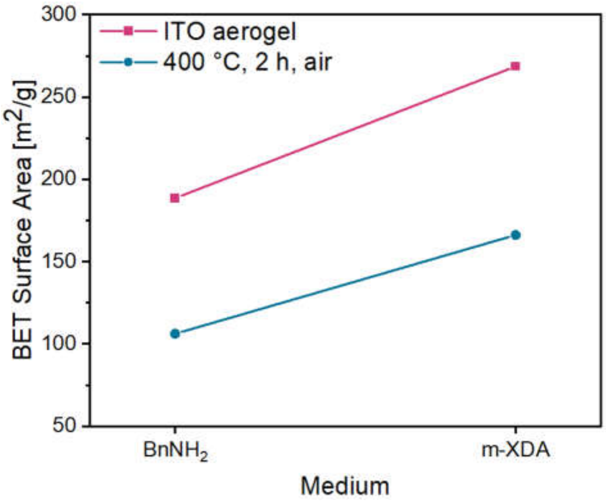

Figure S5. Comparison of the BET surface area of the ITO aerogels after CPD (rose squares) and annealed aerogels in air at 400 °C (blue circles) from the nonaqueous one-pot sol-gel process in $BnNH_2$ and m-XDA.

5. Total pore volume by N_2 gas sorption measurement

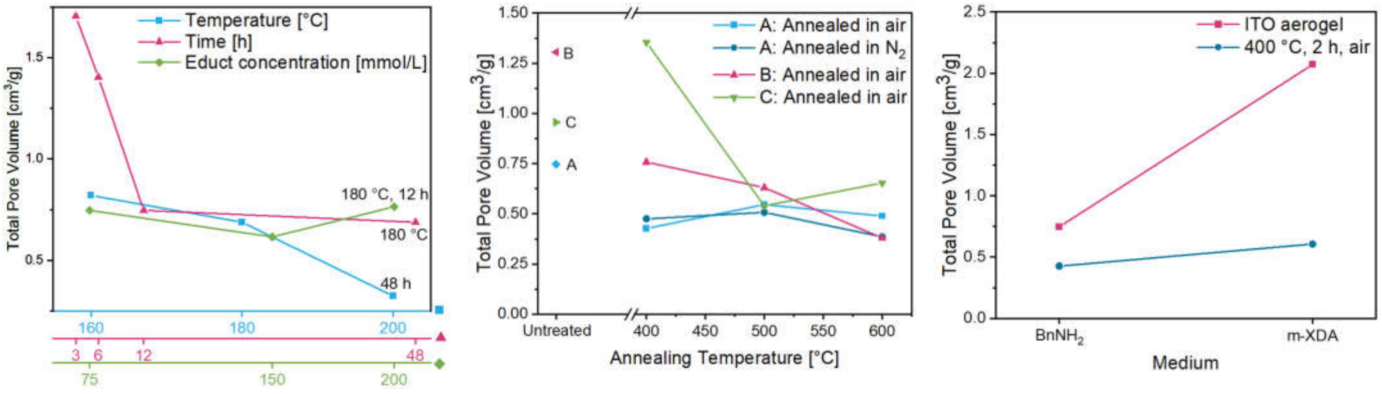

(**a**) (**b**) (**c**)

Figure S6. Total pore volume as a function of different synthesis temperature at a duration of 48 h (blue squares), duration at a temperature of 180 °C (rose triangles), educt concentration of the indium precursor at 180 °C, 12 h (green diamonds) (a). Total pore volume as a function of the ITO aerogel **A** after CPD (blue diamond), **B** (rose left triangle), **C** (green right triangle) and after annealing in air at temperatures from 400 – 600 °C (b). Comparison of the total pore volume from the ITO aerogels before and after annealing at 400 °C in air in the nonaqueous one-pot sol-gel process (c).

6. ATR-IR spectroscopy

The residual surface organics were analyzed with a Bruker ALPHA FT-IR spectrophotometer by attenuated total reflectance infrared (ATR-IR) spectroscopy in the range of 375 – 4000 cm^{-1} wavenumbers. Due to the strong IR absorption properties of ITO, the identification of surface-adsorbed organics via ATR-IR spectroscopy was not possible. Nevertheless, we investigated the supernatant after the MEEAA functionalization treatment and precipitation of the particles. The spectrum of this solution is illustrated in Figure S7 (rose) and has a similar peak pattern as the pure NaCl solution (blue). Since MEEAA is highly diluted in the solution, no representative conclusions can be made regarding its changing property after the addition of NaCl.

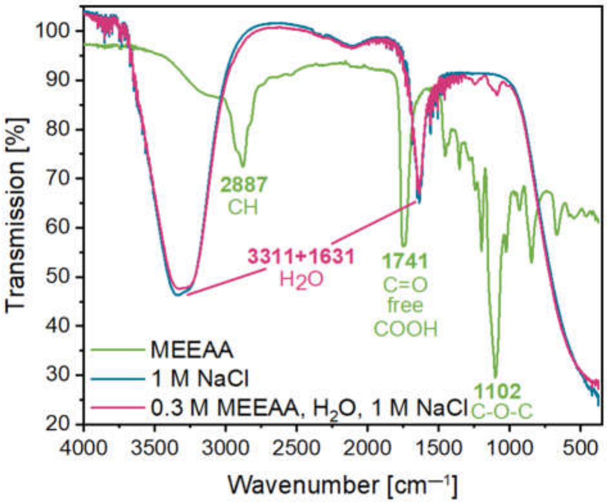

Figure S7. ATR-IR measurements of MEEAA (green), a 1 M NaCl solution (blue) and a solution containing 0.3 M MEEAA, H_2O and— 1 M NaCl (rose).

7. TGA measurements

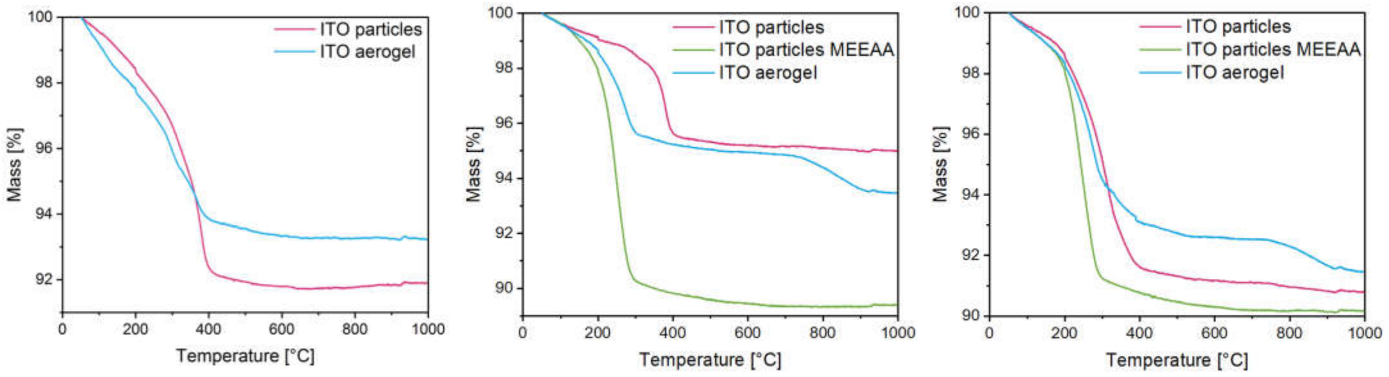

(**a**) (**b**) (**c**)

Figure S8. TGA results of aerogel systems **A** (a), **B** (b) and **C** (c) comparing the respectively obtained ITO particles after direct drying of the as-synthesized product (rose), the ITO nanoparticles after stabilization with MEEAA for systems **B** and **C** (green), and the respective ITO aerogels obtained after CPD (blue).

8. ITO aerogel with m-XDA

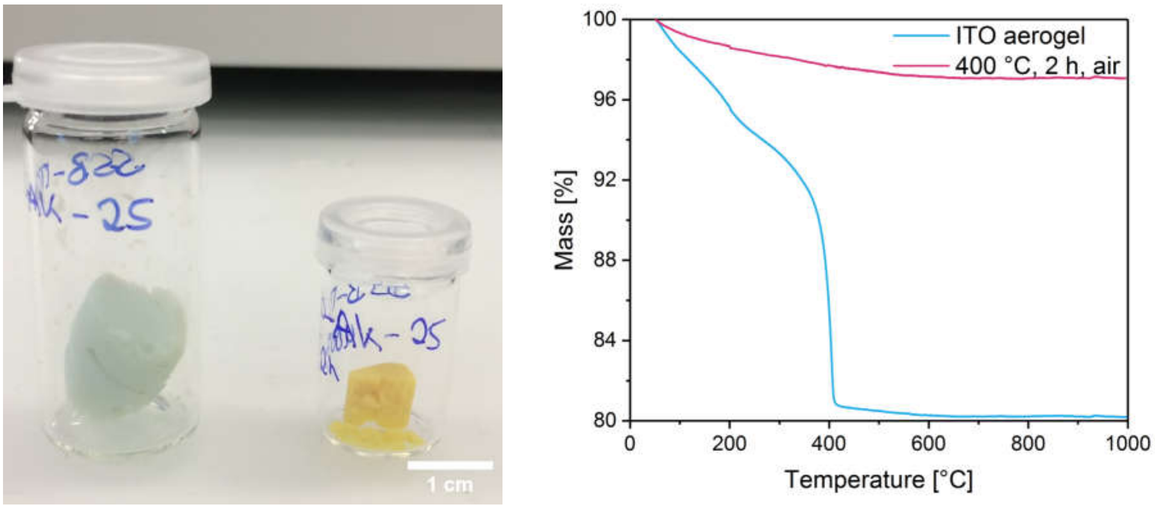

(**a**) (**b**)

Figure S9. ITO aerogel (15 at.% Sn) via the nonaqueous one-pot sol-gel process in m-XDA before (blue) and after annealing at 400 °C in air (yellow) in glass vials (a) and TGA results (b) of the aerogel after CPD (blue) and annealed at 400 °C in air (rose).

9. Pore size distribution by DFT analysis of N_2 gas sorption measurements

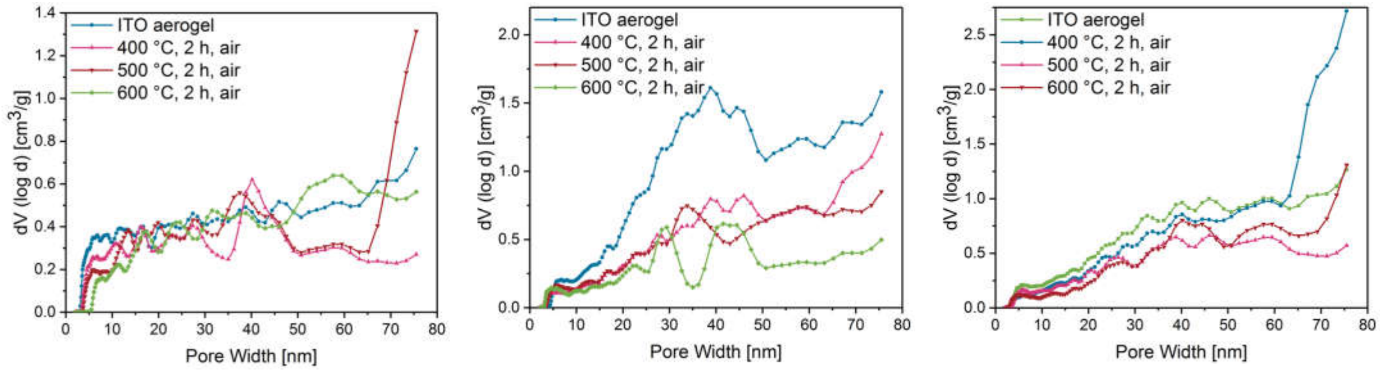

(**a**) (**b**) (**c**)

Figure S10. Pore size distribution (from 2 – 75 nm) by DFT analysis of N_2 gas sorption measurements of ITO aerogel **A** (a), **B** (b) and **C** (c) showing the aerogels directly after CPD (blue) and annealed aerogels at 400 °C (rose), 500 °C (red) and 600 °C (green) in air.

10. BET plots derived from the isotherms in the N_2 gas sorption measurements

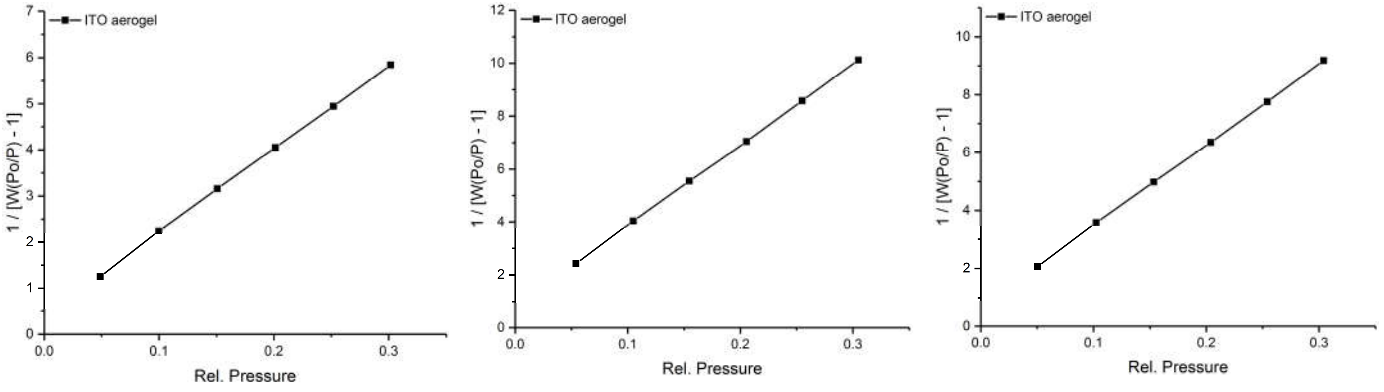

(**a**) (**b**) (**c**)

Figure S11. BET plots derived from the corresponding nitrogen sorption isotherms of ITO aerogels **A** (a), **B** (b) and **C** (c) presented in Figure 11. Good linear fits were obtained in all cases.
